# Dental loss, stress fractures, and musculoskeletal pain in a 48‐year‐old woman

**DOI:** 10.1002/ccr3.7002

**Published:** 2023-03-07

**Authors:** Lindsay T. Hoang, Jennifer S. Hatfield, Quan Nguyen, Mohamed K. M. Shakir, Thanh D. Hoang

**Affiliations:** ^1^ Division of Endocrinology, Department of Medicine Walter Reed National Military Medical Center Bethesda Maryland USA; ^2^ Department of Medicine Memorial Hospital West Pembroke Pines Florida USA; ^3^ Division of Endocrinology, Department of Medicine Uniformed Services University of the Health Services Bethesda Maryland USA

**Keywords:** dental loss, low alkaline phosphatase, stress fracture

## Abstract

We report a 48‐year‐old female patient with various stress fractures of extremities, musculoskeletal pain, and tooth loss. Hypophosphatasia was diagnosed based on clinical and laboratory findings and ALPL genetic results. This case highlights the importance of early diagnosis of hypophosphatasia in adults and appropriate treatment to prevent further complications.

## Graphical abstract

Adult hypophosphatasia may present with recurrent fractures, chronic musculoskeletal pain, tooth loss, and low alkaline phosphatase level. Asfotase alfa therapy can be used to improve respiratory function, motor function, and reduce tooth loss.

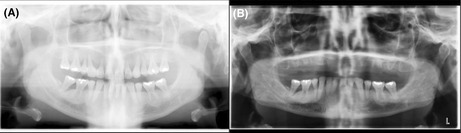

## 1 CASE PRESENTATION

**FIGURE 1:**
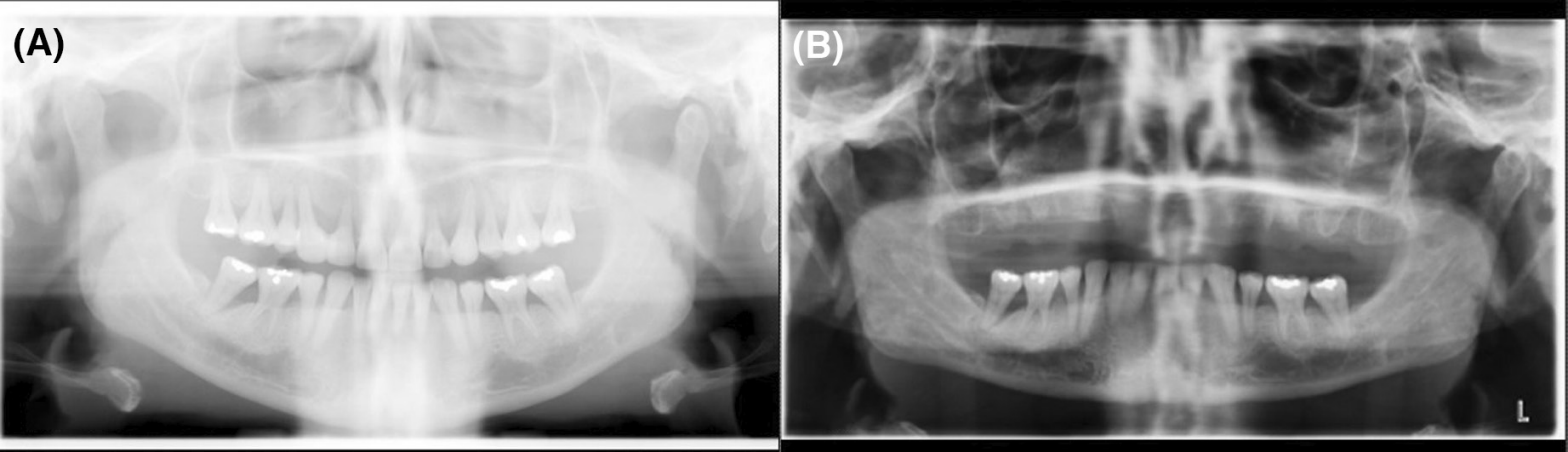
(A and B) Pano pre‐op and post‐op X‐rays of extraction of all upper teeth

**FIGURE 2:**
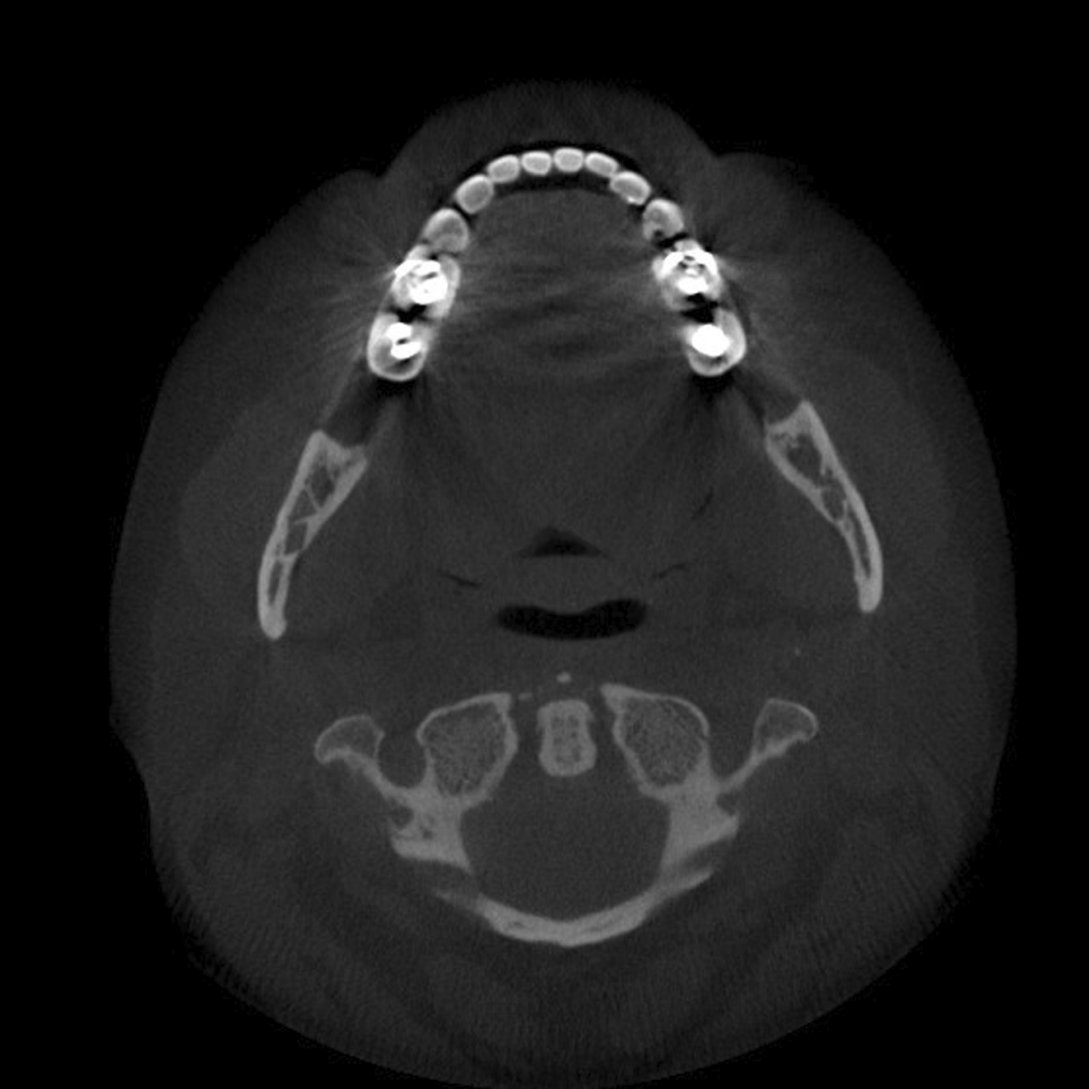
Cone beam CT scan showed edentulous maxilla, thin cortical contours, sclerotic cancellous bone surrounding the teeth and bone loss of mandibular teeth

**FIGURE 3:**
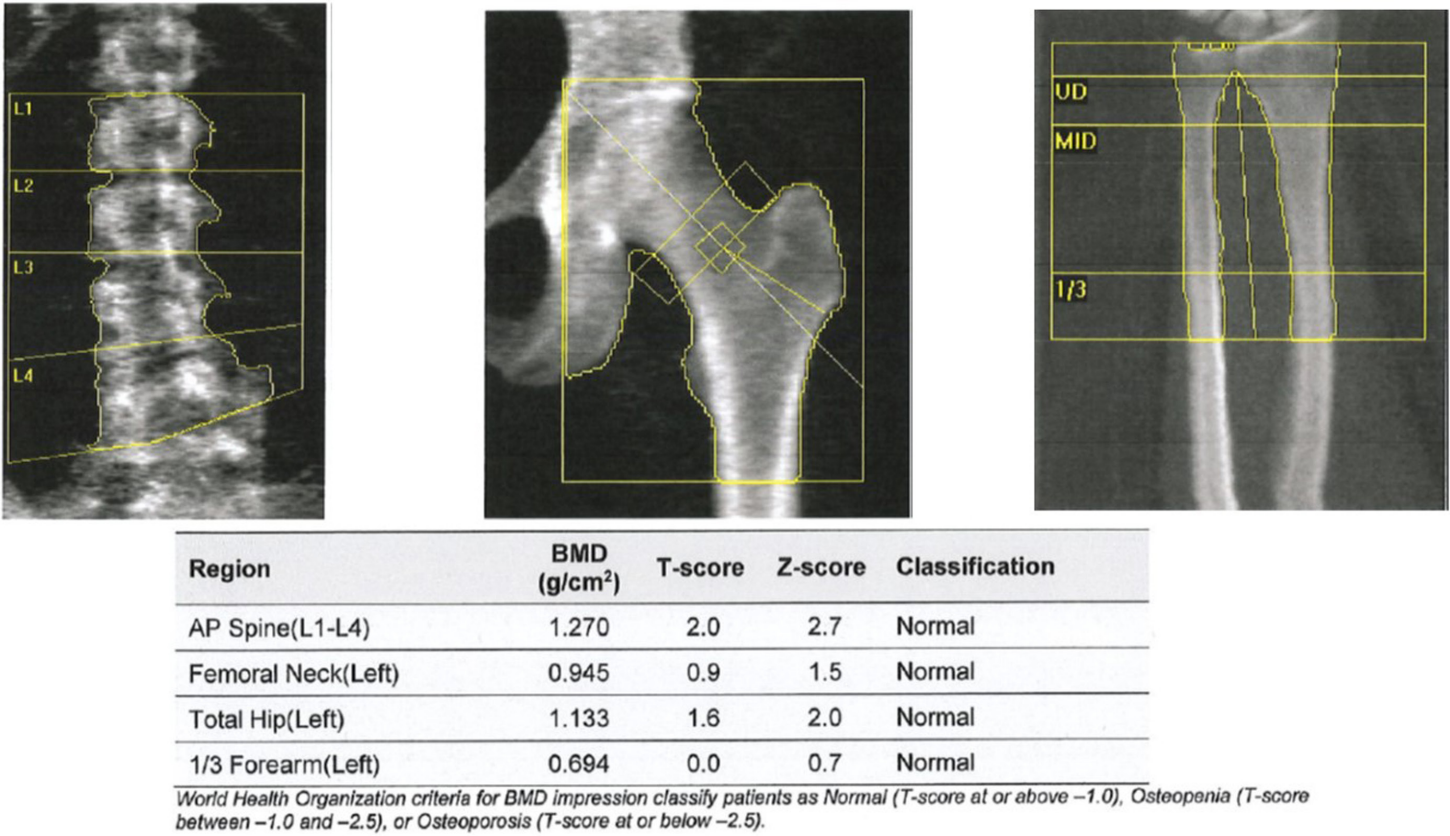
Dual‐energy X‐ray absorptiometry showing normal bone mineral density in the lumbar spine, hip, and forearm

A 48‐year‐old female patient presented with a history of vitamin D deficiency, stress fractures of various extremities, and dental issues with loose teeth. Past medical/surgical history also included perimenopause, nasal cartilage collapse, breast reduction, and 4 bone grafts. She was from European descent, and family history was notable for a mother with early adulthood hip fracture and osteoporosis. She denied any use of tobacco, alcohol, and did not exercise regularly due to pain and limited range of motion. On physical examination, vital signs, heart, and lungs were normal. Dental examination demonstrated loosen teeth of both upper and lower jaws confirmed on X‐rays (Figure 1) and diffuse muscle/joint pain throughout the body. Laboratory findings showed serum alkaline phosphatase level 21 U/L (normal 35–113), normal renal, parathyroid hormone (PTH), and thyroid function tests. Cone beam CT scan showed edentulous maxilla, thin cortical contours, sclerotic cancellous bone surrounding the teeth, and bone loss of mandibular teeth (Figure 2). Genetic testing revealed a single heterozygous pathogenic variant in the ALPL gene. Baseline dual‐energy X‐ray absorptiometry (DXA) was normal (Figure 3).

## 2 WHAT IS THE DIAGNOSIS?

Answer: Hypophosphatasia.

Dental findings and medical history are all consistent with the diagnosis of juvenile onset hypophosphatasia. Hypophosphatasia is characterized by defective mineralization of bone and/or teeth and low serum and bone alkaline phosphatase.^1^, ^2^, ^3^ Severe cases include stillbirth without mineralized bone while milder cases include recurrent fractures of the lower extremities or premature tooth loss in adulthood. Perinatal and infantile hypophosphatasia are inherited in an autosomal recessive manner in which both parents contribute one copy of the mutated gene while adult/milder forms can be inherited in an autosomal dominant or recessive manner where only one copy of the mutated gene is needed for the person to show symptoms. Affected infants may present with short limbs, soft skull bones, and unusual chest shape. Other complications include hypercalcemia, respiratory problems, and difficulty gaining weight. Early primary tooth loss is one of the first signs of this condition in children, whereas adult forms may include recurring fractures, chronic pain, and premature tooth loss of the secondary set of teeth, as in our patient despite normal DXA scan. A characteristic dental finding of hypophosphatasia is early exfoliation of primary teeth, most commonly affecting the anterior teeth, starting between the ages of 1 and 4 years. In hypophosphatasia, early exfoliation of primary teeth is caused by poor mineralization of cementum due to low alkaline phosphatase activity. Disturbed formation of cementum is also recognized in permanent teeth, which may result in permanent teeth being exfoliated. The disturbed formation of cementum increases the susceptibility for early exfoliation of anterior teeth and also ankylosis involving posterior teeth. The occlusal force damages weak periodontal tissue and subsequently interferes with root adhesion to alveolar bone. Thus, early diagnosis is important for patients to receive early intervention in dental fields. In these patients with early exfoliation of primary teeth, dentures are generally recommended so as to ensure that important oral functions are acquired. Studies have shown that treatment with asfotase alfa injection therapy improves dental mineralization, resulting in the stabilization of periodontal tissues and ultimately resulting in better growth of tooth roots.^4^, ^5^, ^6^, ^7^ Treatment with asfotase alfa injection is FDA approved for patients with perinatal/infantile and juvenile‐onset hypophosphatasia and proven to improve respiratory function, motor function, and bone mineralization.^3^ This case highlights the importance of early diagnosis of hypophosphatasia in adults and appropriate treatment to prevent further complications.

## AUTHOR CONTRIBUTIONS

**Lindsay Thi Hoang:** Conceptualization; data curation; formal analysis; investigation; writing – original draft. **Jennifer S Hatfield:** Conceptualization; formal analysis; investigation; writing – review and editing. **Quan Nguyen:** Conceptualization; investigation; writing – review and editing. **Mohamed KM Shakir:** Conceptualization; formal analysis; investigation; resources; supervision; writing – review and editing. **Thanh D Hoang:** Conceptualization; formal analysis; investigation; methodology; resources; supervision; validation; writing – review and editing.

## CONFLICT OF INTEREST STATEMENT

None to declare.

## ETHICS STATEMENT

The manuscript has been reviewed and approved by the IRB and Public Affairs Office.

## INFORMED CONSENT

Written informed consent was obtained from the patient to publish this report in accordance with the journal's patient consent policy.

## Data Availability

Not applicable.
